# Adiposity and mortality in older Chinese: an 11-year follow-up of the Guangzhou Biobank Cohort Study

**DOI:** 10.1038/s41598-020-58633-z

**Published:** 2020-02-05

**Authors:** Chao Qiang Jiang, Lin Xu, Wei Sen Zhang, Ya Li Jin, Feng Zhu, Kar Keung Cheng, Tai Hing Lam

**Affiliations:** 1 Guangzhou No.12 Hospital, Guangzhou, 510620 China; 2 School of Public Health, Sun Yat-Sen University, Guangzhou, China; 3 School of Public Health, the University of Hong Kong, Hong Kong, China; 4 Institute of Applied Health Research, University of Birmingham, Birmingham, UK

**Keywords:** Obesity, Risk factors

## Abstract

Previous studies on Chinese showed mixed results describing the relationship between obesity and mortality. The optimum levels of body mass index (BMI) and waist circumference (WC) are inconsistent. In the Guangzhou Biobank Cohort Study, after excluding ever smokers and those with poor health, 19,405 Chinese (50+ years) recruited from 2003 to 2008 were followed-up until 2017. During an average follow-up of 11.5 (standard deviation = 2.3) years, 1,757 deaths were recorded. All-cause mortality showed a J-shaped association with BMI, with the lowest mortality risks at 22.5 kg/m^2^ for both men and women. In those with BMI ≥ 22.5 kg/m^2^, an increase of 5 kg/m^2^ was associated with 29% higher all-cause mortality (hazard ratio (HR) = 1.29, 95% confidence interval (CI) 1.15–1.46), 30% higher cancer mortality (1.30, 95% CI 1.08–1.57), and 37% higher cardiovascular disease (CVD) mortality (1.37, 95% CI 1.13–1.67) after adjustment for potential confounders. In this first cohort study in one of the most economically developed cities in China, the lowest all-cause mortality was observed for a BMI of 22.5 kg/m^2^ in all participants, and a WC of 78 cm in men and 72 cm in women.

## Introduction

The global emergence of the obesity epidemic has serious adverse health effects. WHO Global Health Observation data shows that at least 2.8 million deaths and 35.8 million of Disability Adjusted Life Years are attributable to adiposity each year globally^1^. Most of the estimates above are based on studies in high-income countries several decades ago. As developing countries have only recently experienced a short period of obesity, the adverse population effects on morbidity or mortality have not been evident and tend to be underestimated. The effect in the low-to-middle income and high-income countries needs to be examined separately and the disease burden updated urgently.

China is now in the first stage of the obesity epidemic^2^, as indicated by a relatively low prevalence of obesity^3,4^ and obesity attributable mortality risk^4–6^. Although the prevalence is increasing rapidly^7^, its effect on mortality has yet to be elucidated. Moreover, previous studies of obesity and mortality did not comprehensively account for pre-existing illnesses, which, if ignored, may result in reverse causality. For example, unmeasured underlying disease reduces body weight and leads to an observation that lower body weight was detrimental and obesity was protective^8–11^. Additionally, the obesity-mortality association is often confounded by smoking, and simply adjusting for smoking status does not eliminate such confounding^12^. Studies failing to account for reverse causality and smoking might underestimate the excess risk due to overweight and obesity especially in never smokers^13^. Note that previous studies of Chinese populations did not show an adverse effect of overweight or obesity (i.e., BMI ≥ 24 or 28 kg/m^2^, respectively) on all-cause and cause-specific mortality^9–11,14,15^.

Although useful on a population level, for individuals BMI has some limitations due to its low discrimination of fat distribution. WC has also been used as a simple surrogate marker for central adiposity, yet the optimal WC varies across different ethnicities. Due to these uncertainties in the adiposity-mortality association and the optimal BMI and WC levels in Chinese, we used data from the Guangzhou Biobank Cohort Study (GBCS), a well-established population-based cohort of 30,000 people living in Guangzhou, China to examine the association between adiposity and mortality, accounting for reverse causation and smoking, and to identity the levels of BMI and WC with the lowest mortality risk.

## Subjects and Methods

### Study subjects

All participants of the GBCS were recruited from 2003 to 2008. Details of the GBCS have been reported elsewhere^16,17^. Briefly, all participants of the GBCS was from “The Guangzhou Health and Happiness Association for the Respectable Elders” (GHHARE). GHHARE is a community social and welfare organization unofficially aligned with the Guangzhou government. Membership is open to local residents aged 50+ years for a minimal nominal fee (sabout 50 US cents) per month. GHHARE included about 7% of residents in this age group, with branches in all districts of Guangzhou, a mega city in southern China. The baseline examination included a computer-based face-to-face interview by nurses. Information of demographic characteristics, lifestyle factors, family and personal medical history, and detailed assessment of anthropometrics, blood pressure, fasting plasma glucose, lipids and inflammatory markers was collected.

### Adiposity measures

Anthropometric measures including standing height, body weight and WC were measured with light indoor clothing and no shoes according to a standard protocol. Height was measured to the nearest 0.1 cm and weight was measured to the nearest 0.1 kg^16^. BMI was calculated as weight divided by height squared (kg/m^2^). The Chinese-specific cut-offs for general adiposity, as per the World Health Organization (WHO) recommendation, were used, with underweight defined as BMI lower than 18.5 kg/m^2^, normal weight as BMI 18.5–24.9 kg/m^2^, overweight as BMI 25.0–27.4 kg/m^2^ and general obesity as BMI ≥ 27.5 kg/m^2^^18^. According to Asian-specific cut-offs suggested by WHO, central obesity was defined by WC ≥ 90 cm in men and ≥80 cm in women^19^. Waist circumference (WC) was measured to the nearest 0.1 cm, using a non-stretch tape horizontally around the narrowest part of the torso between the lowest rib and the iliac crest.

### Mortality

Information on underlying causes of deaths up to December 2017 was obtained via record linkage with the Death Registry of the Center for Disease Control and Prevention (CDC) in Guangzhou. Causes of death were coded by trained nosologists in each hospital. Causes of death were verified by CDC as part of their quality assurance programme by cross-checking past medical history and conducting verbal autopsy when the death certificates were not issued by medical institutions. Ten verbal autopsy meetings were conducted in the Guangzhou 12^th^ Hospital to verify the deaths with uncertain causes. Coding of the endpoints in this study was shown in the Supplementary Table 1.

### Statistical analysis

Analysis of variance or Chi-square tests were used to compare baseline characteristics by BMI/WC groups. Associations between BMI/WC and mortality were estimated by Cox regression giving hazard ratios (HRs) and 95% confidence intervals (CI). Potential confounders including age (as continuous), sex (except for breast cancer mortality), education (primary and below, secondary school, and college or above), occupation (manual, non-manual, and others), family income (<30,000 CNY/year, ≥30,000 CNY/year, and not known; US$1 = 7 CNY), International Physical Activity Questionnaire assessed physical activity (inactive, moderate and active)^20^, alcohol use (never, former, and current drinkers) and self-rated health (very good, good, poor and very poor). To limit biases that could be caused by underlying illnesses, the main analysis was done by excluding participants who reported poor health status and were ever smokers (including current and former smokers) at baseline. Poor health status at baseline was defined as any of the following conditions: (1) regular use of medication for chronic diseases, such as diabetes, hypercholesterolaemia, or vascular diseases, or (2) any hospital admission during past 6 months, or (3) self-reported cardiovascular disease history, or (4) self-reported cancer history^3^. Sensitivity analyses were also done in all participants (including ever and current smokers as well as those with poor health), as in most other studies^5,6,21^. All analysis was performed by STATA/IC 16.0.

### Ethical considerations

This study was approved by the Guangzhou Medical Ethics Committee of the Chinese Medical Association. All participants signed informed consent before participation. The study was performed in accordance with the Declaration of Helsinki.

## Results

At baseline, of the 30,430 participants, 391 lost to follow-up and 150 did not measure BMI or WC were excluded, giving 29,981 participants (21,698 women and 8,283 men). Of them, 5,830 participants who were former or current smokers, and 4,746 participants with poor health were excluded from the main data analysis of this paper, giving 16,840 women and 2,565 men. The mean (standard deviation) age of 19,405 participants was 62 (7.1) years. During the average follow-up of 11.5 (SD = 2.3) years or 225,867 person-years, 1,757 (women 1,371 (8.2%) and men 386 (15.1%)) deaths were recorded.

Most (62.4%) participants had a normal BMI of 18.5 to <25 kg/m^2^; 4.4% had a lower BMI (<18.5 kg/m^2^) and 33.2% had a greater BMI of ≥25 kg/m^2^ (Table 1). Participants with a lower BMI were older, more likely to be men, had higher education, and less likely to have lower manual occupation, be alcohol users and in good self-reported health (all P < 0.01) (Table 1). Similarly, participants with higher WC was older, less likely to be men, had lower education, more likely to have manual occupation and higher family income, be physically active and in good self-rated health (all P < 0.01, Table 1).

**Table 1:** Baseline characteristic of 19,405 participants aged 50+ in the Guangzhou Biobank Cohort Study first examined in 2003 to 2008 and followed up until January 2016, after excluding ever smokers and those with poor health.

	BMI, kg/m^2^							
	<18.5	18.5 to <20	20 to <22.5	22.5 to <25	25 to <27.5	27.5 to <30	30+	P value
Number of participants (row %)	853 (4.4)	1414 (7.3)	4745 (24.5)	5940 (30.6)	4040 (20.8)	1703 (8.8)	710 (3.7)	
Age, years, mean (SD)	62.5 (7.5)	61.3 (7.3)	60.6 (7.2)	60.9 (7)	61 (6.8)	61.1 (6.9)	60.7 (6.5)	<0.001
Sex, % men	14.1	12.1	12.1	14.2	14.7	12.3	8.0	<0.001
Education, % Primary or below	43.7	41.3	39.0	42.3	46.8	51.2	57.2	<0.001
Occupation, % Manual	64.7	63.9	61.7	63.1	64.6	67.0	69.0	<0.001
Family income (CNY/year), % < 30,000	37.0	37.7	36.6	35.6	37.4	37.4	35.4	<0.001
Physical activity, % Active	50.6	53.8	54.7	53.2	52.7	50.9	52.5	0.10
Alcohol use, % Current	14.9	18.5	20.1	21.1	22.1	21.2	21.6	0.003
Self-rated health, % good	74.1	79.7	85.1	86.8	86.7	85.7	85.5	<0.001
	**Waist circumference, cm**							
	**<70 for M** **<65 for F**	**70 to <75 for M** **65 to <70 for F**	**75 to <80 for M** **70 to <75 for F**	**80 to <85 for M** **75 to <80 for F**	**85 to <90 for M** **80 to <85 for F**	**90 to <95 for M** **85 to <90 for F**	**95+ for M** **90+ for F**	**P value**
Number of participants (row %)	1341 (6.9)	2382 (12.3)	3991 (20.6)	4400 (22.7)	3671 (18.9)	2075 (10.7)	1545 (8.0)	—
Age, years, mean (SD)	60 (7.1)	59.5 (7.1)	59.9 (6.9)	60.7 (6.9)	61.7 (6.9)	62.7 (6.9)	63.1 (7)	< 0.001
Sex, % men	17.5	11.8	11.8	13.8	13.9	14.5	10.5	<0.001
Education, % Primary or below	33.9	34.1	36.7	41.2	49.4	56.0	62.7	<0.001
Occupation, % Manual	59.1	59.5	60.5	63.6	65.6	69.4	71.4	<0.001
Family income (CNY/year), % < 30,000	35.7	35.9	35.1	35.3	37.2	39.7	40.1	<0.001
Physical activity, % Active	50.9	53.2	52.8	53.7	52.5	52.5	57.0	<0.001
Alcohol use, % Current	21.3	21.5	21.7	20.7	20.5	18.8	18.5	0.18
Self-rated health, % good	78.0	83.9	85.2	86.8	86.4	85.0	85.5	<0.001

### BMI and mortality

After excluding current and former smokers and those with poor health status, BMI showed a J-shaped association with the risk of all-cause mortality, with the lowest mortality risks at a BMI of 20 to <22.5 kg/m^2^. Compared with participants with BMI of 20 to <22.5 kg/m^2^, and with adjustment of age, sex, occupation, personal income, physical activity, alcohol use and self-rated health, participants with BMI of 27.5 kg/m^2^ and above (i.e., meeting the definition of obesity in China) had a 38% greater risk for all-cause mortality (HR 1.38, 95% CI 1.17–1.62), a 50% greater risk for cardiovascular disease mortality (HR 1.50, 95% CI 1.14–1.97), a 65% greater risk for ischemic heart disease (IHD) mortality (HR 1.65, 95% CI 1.06–2.56) and a 37% greater risk for cancer mortality (HR 1.37, 95% CI 1.06–1.78) (Table 2).

**Table 2:** Adjusted hazards ratio (AHRs) for all-cause and cause-specific mortality by body mass index (BMI) recruited during 2003-8 and followed up till December 2017, after excluding ever smokers and those with poor health.

	BMI. Kg/m^2^						BMI ≥ 22.5 kg/m^2^
	<18.5	18.5 to <20	20 to <22.5	22.5 to <25	25 to <27.5	≥27.5	Per 5 kg/m2
Person years	9840	16266	55438	69418	47067	27838	142962
**All-cause**							
No. of deaths	109	150	381	508	357	252	1108
AHR (95% CI)^†^	1.35 (1.09–1.68)**	1.26 (1.04–1.53)*	Ref.	1.06 (0.92–1.21)	1.14 (0.98–1.32)	1.38 (1.17–1.62)***	1.29 (1.15–1.46)***
**All cancer**							
No. of deaths	109	150	381	508	357	252	1108
AHR (95% CI)^†^	1.2 (0.83–1.73)	1.11 (0.81–1.54)	Ref.	1.05 (0.85–1.3)	1.00 (0.78–1.27)	1.37 (1.06–1.78)*	1.30 (1.08–1.57)**
**Liver cancer**							
No. of deaths	4	5	18	32	15	11	57
AHR (95% CI)^†^	1.1 (0.37–3.29)	0.99 (0.36–2.72)	Ref.	1.55 (0.84–2.85)	1.16 (0.57–2.34)	1.51 (0.7–3.26)	1.00 (0.57–1.77)
**Colorectal cancer**							
No. of deaths	10	3	27	26	21	18	65
AHR (95% CI)^†^	1.9 (0.91–3.96)	0.39 (0.12–1.28)	Ref.	0.77 (0.44–1.35)	1.01 (0.57–1.81)	1.51 (0.82–2.78)	1.83 (1.19–2.82)**
**Cardiovascular disease**							
No. of deaths	36	59	126	174	131	90	394
AHR (95% CI)^†^	1.35 (0.93–1.95)	1.53 (1.12–2.08)**	Ref.	1.1 (0.87–1.39)	1.26 (0.99–1.62)	1.50 (1.14–1.97)**	1.37 (1.13–1.67)**
**IHD**							
No. of deaths	19	26	45	66	53	37	155
AHR (95% CI)^†^	1.85 (1.07–3.2)*	1.86 (1.15–3.01)*	Ref.	1.16 (0.79–1.7)	1.42 (0.95–2.13)	1.65 (1.06–2.56)*	1.35 (0.98–1.86)
**Stroke**							
No. of deaths	15	27	62	75	53	38	166
AHR (95% CI)^†^	1.17 (0.67–2.07)	1.43 (0.91–2.25)	Ref.	0.97 (0.69–1.37)	1.04 (0.71–1.5)	1.35 (0.9–2.02)	1.37 (1.01–1.85)*
**Respiratory disease**							
No. of deaths	17	15	43	54	34	27	114
AHR (95% CI)^†^	1.82 (1.02–3.25)*	1.09 (0.58–2.03)	Ref.	1.05 (0.7–1.58)	1.04 (0.66–1.65)	1.49 (0.91–2.43)	1.35 (0.93–1.96)
**Diabetes**							
No. of deaths	1	1	3	5	8	7	20
AHR (95% CI)^†^	—	—	Ref.	1.1 (0.24–4.92)	3.06 (0.81–11.62)	4.88 (1.25–19.02)*	2.47 (1.22–4.98)*

CI: confidence interval; IHD: ischemic heart disease.

^†^Adjusted for age, sex, occupation, personal income, physical activity, alcohol use and self-rated health.

*P < 0.05; **P < 0.01; ***P < 0.001.

**Figure 1:**
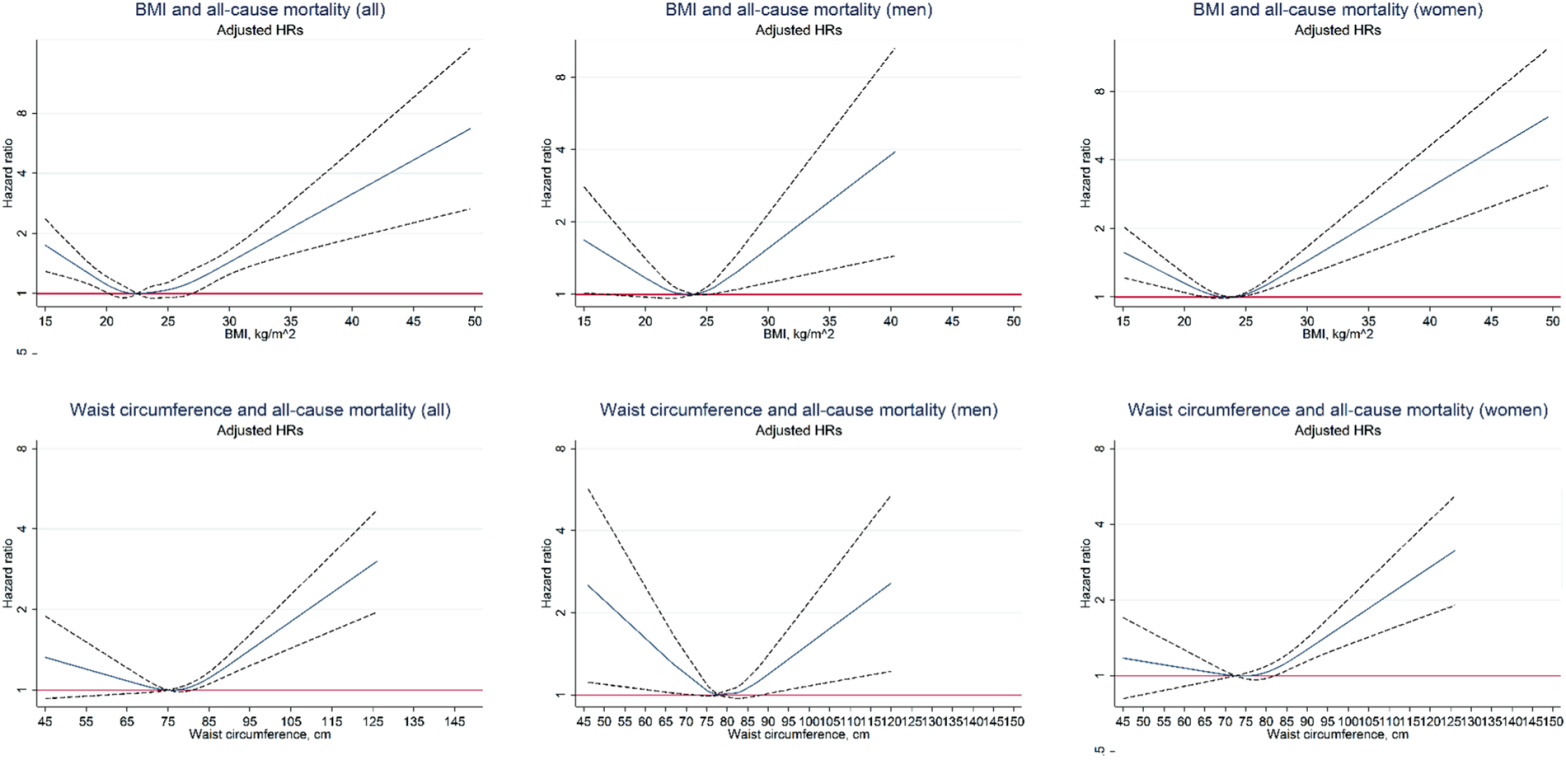
Association of body mass index (BMI) and waist circumference with all-cause mortality in 19,405 participants of the Guangzhou Biobank Cohort Study recruited during 2003–8 and followed up till December 2017, after excluding ever smokers and those with poor health status. All hazard ratios were adjusted for age, sex, occupation, personal income, physical activity, alcohol use and self-rated health.

Figure 1 shows that, BMI in men and women at about 22.5 kg/m^2^ and WC at about 78 cm in men and 72 cm in women showed the lowest risk for all-cause mortality. Table 2 shows that in those with a BMI ≥ 22.5 kg/m^2^, an increase of 5 kg/m^2^ was associated with 29% greater all-cause mortality (HR 1.29, 95% CI 1.15–1.46), 37% higher CVD mortality (HR 1.37, 95% CI 1.13–1.67), 37% higher stroke mortality (HR 1.37, 95% CI 1.01–1.85), 30% higher all cancer mortality (HR 1.30, 95% CI 1.08–1.57) and 83% higher colorectal cancer mortality (HR 1.83, 95% CI 1.19–2.82). Further adjusting for WC or excluding those aged >  = 85 years showed higher risks for low BMI but lower risks for high BMI (Supplementary Tables 2 and 3). Using the conventional Chinese-specific cut-offs for general obesity status, relative to normal weight (18.5 ≤ BMI < 25 kg/m^2^), general obesity (BMI ≥ 27.5 kg/m^2^) was associated with 30% (HR 1.30, 95% CI 1.13–1.50) greater risk of al-cause mortality, 35% greater risk of CVD mortality (HR 1.35, 95% CI 1.07–1.70), 32% greater risk of all cancer mortality (HR 1.32, 95% CI 1.06–1.65), and almost 2-fold risk of colorectal cancer (HR 1.86, 95% CI 1.09–3.18) (Supplementary Table 4). After adjustment for potential confounders related to baseline BMI groups (Supplementary Table 5), the patterns for BMI groups remained in all 29,981 participants including those who were smokers and with poor health, with slightly attenuated hazard ratios (Supplementary Table 6).

### WC and mortality

In men with WC of 78+ cm and women with WC of 72+ cm, after adjusting for age, sex, occupation, personal income, physical activity, alcohol use and self-rated health, each 10 cm greater WC was associated with 18% higher all-cause mortality (adjusted HR 1.18, 95% CI 1.10–1.27), 16% greater risk of CVD mortality (adjusted HR 1.16, 95% CI 1.03–1.30), 17% higher cancer mortality (adjusted HR 1.17, 95% CI 1.04–1.32) and 98% higher diabetes mortality (adjusted HR 1.98, 95% CI 1.20–3.27) (Table 3).

**Table 3:** Adjusted hazards ratio (AHRs) for all-cause and cause-specific mortality by waist circumference recruited during 2003–8 and followed up till December 2017, after excluding ever smokers and those with poor health.

	Waist circumference, cm							78+ for M and 72+ for M; Per 10 cm
	<70 for M < 65 for F	70 to <75 for M 65 to <70 for F	75 to <80 for M 70 to <75 for F	80 to <85 for M 75 to <80 for F	85 to <90 for M 80 to <85 for F	90 to <95 for M 85 to <90 for F	95+ for M 90+ for F	
Person years	15433	27336	46388	51682	42903	24311	17815	183098
**All-cause**								
No. of deaths	108	179	300	337	363	249	221	1470
AHR (95% CI)^†^	1.01 (0.81–1.26)	1.02 (0.84–1.23)	Ref.	0.89 (0.76–1.04)	1.07 (0.92–1.25)	1.16 (0.98–1.38)	1.41 (1.18–1.68)***	1.18 (1.1–1.27)***
**All cancer**								
No. of deaths	38	72	122	145	121	82	87	557
AHR (95% CI)^†^	0.93 (0.64–1.34)	1.04 (0.77–1.4)	Ref.	1 (0.78–1.28)	0.98 (0.76–1.27)	1.1 (0.82–1.46)	1.61 (1.21–2.13)**	1.17 (1.04–1.32)**
**Liver cancer**								
No. of deaths	4	6	12	24	20	9	10	75
AHR (95% CI)^†^	0.95 (0.3–3)	0.77 (0.27–2.21)	Ref.	1.6 (0.77–3.3)	1.68 (0.8–3.51)	1.15 (0.48–2.8)	1.76 (0.74–4.18)	1.04 (0.75–1.45)
**Colorectal cancer**								
No. of deaths	4	10	23	23	17	12	16	91
AHR (95% CI)^†^	0.55 (0.19–1.59)	0.76 (0.35–1.66)	Ref.	0.95 (0.53–1.72)	0.81 (0.43–1.55)	0.96 (0.47–1.95)	1.81 (0.94–3.51)	1.21 (0.91–1.61)
**Cardiovascular disease**								
No. of deaths	34	59	107	106	141	92	77	523
AHR (95% CI)^†^	0.89 (0.6–1.31)	0.92 (0.67–1.27)	Ref.	0.76 (0.58–0.99)*	1.09 (0.85–1.41)	1.09 (0.82–1.45)	1.23 (0.92–1.66)	1.16 (1.03–1.3)*
**IHD**								
No. of deaths	16	25	43	41	48	38	35	205
AHR (95% CI)^†^	1.01 (0.56–1.83)	0.96 (0.59–1.58)	Ref.	0.72 (0.47–1.1)	0.9 (0.59–1.36)	1.09 (0.7–1.7)	1.37 (0.87–2.15)	1.18 (0.98–1.42)
**Stroke**								
No. of deaths	18	26	49	44	69	33	31	226
AHR (95% CI)^†^	1.03 (0.6–1.77)	0.87 (0.53–1.4)	Ref.	0.67 (0.44–1.01)	1.16 (0.8–1.68)	0.84 (0.54–1.32)	1.07 (0.68–1.69)	1.10 (0.92–1.32)
**Respiratory disease**								
No. of deaths	15	16	32	35	38	28	26	159
AHR (95% CI)^†^	1.21 (0.64–2.27)	0.71 (0.38–1.33)	Ref.	0.81 (0.5–1.3)	0.95 (0.59–1.54)	1.18 (0.71–1.96)	1.47 (0.87–2.48)	1.23 (0.99–1.52)
**Diabetes**								
No. of deaths	2	0	4	2	6	5	6	23
AHR (95% CI)^†^	1.46 (0.26–8.01)	-	Ref.	0.4 (0.07–2.17)	1.2 (0.34–4.27)	1.23 (0.31–4.98)	2.38 (0.66–8.56)	1.98 (1.2–3.27)**

CI: confidence interval; IHD: ischemic heart disease.

^†^Adjusted for age, sex, occupation, personal income, physical activity, alcohol use and self-rated health.

*P < 0.05; **P < 0.01; ***P < 0.001.

Sensitivity analyses showed that the HRs for WC groups attenuated slightly in all 29,981 participants including those who were ever smokers and with poor health (Supplementary Tables 7) Individuals who were classified as having central obesity were associated with an 18% greater risk of all-cause mortality (HR 1.18, 95% CI 1.07–1.30) (Supplementary Table 4). Changing the reference group to those with the lowest WC (Supplementary Table 8), or further adjusted for BMI or excluding participants with BMI < 18.5 kg/m^2^ showed similar results (Supplementary Tables 9 and 10).

## Discussion

In this well-characterised population-based cohort of middle-aged to older Chinese, greater BMI and WC were associated with increased risk of all-cause mortality. The lowest all-cause mortality was among those with a BMI of 22.5 kg/m^2^, with no difference between men and women; the optimal WC varied by sex, being about 78 cm in men, and 72 cm in women. Adiposity above the optimum levels was associated with greater excess mortality risks, with dose response associations, for cardiovascular disease, cancer and stroke. The excesses were slightly smaller when current and former smokers and those with ill health were included, suggesting that reverse causality would lead to under-estimates of the excess risks.

The optimal BMI level of 22.5 kg/m^2^ in our study was lower than those of previous reports from China showing an optimal level of about 25 kg/m^2^ ^2,6,9^. A previous meta-analysis including mostly studies from developed countries and decades ago^21^ or studies from China^6,22^ suggested individuals with overweight or grade 1 obesity (BMI 30–35 kg/m^2^) had a lower risk of all-cause mortality than those with normal BMI. For example, a recent large cohort study in China including more than 120,000 Chinese workers found a greater mortality risk in people with low BMI (<18.5 kg/m^2^) but no association for people with overweight (BMI ≥ 25 kg/m^2^) or general obesity (BMI ≥ 28 kg/m^2^), compared to normal weight (BMI 18.5–24.9)^9^. This study, as some other previous studies^23–26^, did not exclude those with pre-existing disease and smoking^9^, which might mask the BMI-mortality risk association. Moreover, inappropriate adjustment for mediating factors for the associations between BMI and major health endpoints (i.e., CVD), such as glycaemia, triglycerides and low-density lipoprotein cholesterol will attenuate the result towards the null^9,27^. One recent study in China also showed no association between greater BMI groups (i.e., BMI > 26, 28 or 30 kg/m^2^) and all-cause mortality, in subgroups by glycaemic status or in the whole sample^15^. Another recent study showed that after excluding participants with baseline metabolic abnormalities (hypertension, diabetes and/or dyslipidaemia), those with greater BMI (>28 kg/m^2^) had a greater risk of all-cause and CVD mortality, although the results were not statistically significant (HR 1.56, 95% CI 0.85–2.86 and 1.40, 95% CI 0.56–3.51)^14^. Our study, being the first in China to have excluded current and former smokers and those with poor health from a developed city in China, where the obesity epidemic may have progressed to a more advanced stage^3^, clearly showed that greater BMI (>27.5 kg/m^2^) was associated with a greater risk of all-cause, cancer and CVD mortality. Moreover, in our study, a 5 kg/m^2^ greater BMI was significantly associated with 37% risk of CVD mortality. This finding is well consistent with a recent large Mendelian randomization study showing a SD increase (1 SD = 4.5 kg/m^2^) in BMI was causally associated with 44% greater risk of CHD^27^.

Overweight and obesity have been associated with an increased risk of total cancer, where a 5 kg/m^2^ greater BMI is typically associated with a greater risk by about 30%^13,28^. However, the evidence showing adiposity causes cancer, to date, has come mainly from Caucasian populations^28^, with few data from Asians in whom body fat proportions can be substantially greater for a given BMI^29,30^. We found only a single prospective cohort study in Chinese men reporting no association between BMI and cancer in those with BMI of 23.5 kg/m^2^ or greater (HR 0·94, 95% CI 0·76–1·16)^5^. The discrepancies of the BMI-cancer association in different settings may be partly explained by differences in the prevalence of lifestyle factors (i.e., smoking and diet) and sex hormone in different ethnicities^31^. For example, meta-analyses of the Asia-Pacific Cohort Studies Collaboration (APCSC)^13^ and a few Asian studies showed that, elevated BMI was associated with greater risks of colorectal cancer, but not some sex hormone-related cancer, such as prostate cancer in populations from Asia^32–34^. In a study from China, greater BMI was associated with a greater risk of colorectal cancer in men but not women^34^. The authors suggested that mechanisms underlying this sex difference could be related to sex steroid hormone exposures^34^. Other large cohort studies in China did not report a significant association between obesity and colorectal cancer, probably due to the short duration of obesity (i.e., almost all Chinese were thin before the Open Door Policy since 40 years ago) in subjects included in the previous studies^5,6^. Our results support the statement from the World Cancer Research Fund that adiposity was causally associated with greater risk of colorectal cancer mortality^5,6^. Furthermore, our study demonstrated a significantly increased risk of cancer mortality from greater BMI. Our results indicate that the statement that obesity is “only a negligible cause of cancer overall”^5^ in China needs to be reviewed and updated. We did not have enough events to analyse prostate cancer as few deaths from prostate cancer were observed in our cohort.

Our study had several limitations. The first limitation was that only baseline measures of adiposity were used in the current analysis. Information of weight fluctuation (i.e., weight gain or weight loss during the follow-up) and obesity duration was not included. Second, as all participants in our study were aged 50 years or above, generalizability of the results to younger populations, or to non-Chinese may be limited. Third, our sample size is not large compared to other studies on obesity and mortality conducted mainly in the West^12,21^. However, with sufficient follow-up and carefully accounting for reverse causality and confounding, we were able to demonstrate that, in such a less obese population with a relatively short obesity duration, obesity-induced mortality risk has been substantial and become comparable to the more developed settings^12^, suggesting the obesity epidemic is expanding and may have come to an advanced stage in large cities in China (i.e., Guangzhou) before an obvious epidemic can be observed in the whole country. While we expect further follow up of our cohort and future studies may show greater excess risks from adiposity with the expanding of the obesity epidemic^2^, our results highlight that previous studies could have under-estimated the risks, and the cut-off values for over-weight and obesity for Chinese need to be reviewed urgently to support public health actions for obesity control. Our results also forewarn the expanding obesity epidemic in other rapidly developing countries in Asia and elsewhere. Strengths of this study included large sample size compared with other studies in Asia, long follow-up duration, comprehensive assessment of adiposity and lifestyle factors, accurate information about the causes of deaths, and the city-wide representative sample which should have minimized selection bias.

In conclusion, this first cohort study on non-smoking healthy Chinese in one of the most economically developed city in China found that greater BMI or WC was associated with increased risk of all-cause mortality. The lowest all-cause mortality was observed at a BMI of 22.5 kg/m^2^ in all participants, and a WC of 78 cm in men and 72 cm in women. Our results showed that the obesity epidemic in China, which is still at an early stage, may have come to a more advanced stage in large cities. Many other low- and middle-income countries are developing rapidly and our results can forewarn the expanding obesity epidemic and the related disease burden, especially in large cities. Urgent and more stringent actions are needed to halt the epidemic of obesity in the China and globally.

## Supplementary information

Supplementary information

## Data Availability

Due to ethical restrictions protecting patient privacy, data available on request from the Guangzhou Biobank Cohort Study Data Access Committee. Please contact us at gbcsdata@hku.hk for fielding data accession requests.
