# Supplementary material for: Adiposity and mortality in older Chinese: an 11-year follow-up of the Guangzhou Biobank Cohort Study
Source: Sci Rep. 2020 Feb 5;10:1924. doi: 10.1038/s41598-020-58633-z (PMC7002501; doi:10.1038/s41598-020-58633-z)
Supplement: Supplementary file 1 — Supplementary information [file 41598_2020_58633_MOESM1_ESM.docx]

**Adiposity and mortality in older Chinese: an 11-year follow-up of the Guangzhou Biobank Cohort Study**

Chao Qiang Jiang, *MD* ^1^, Lin Xu, *PhD* ^2,3*^, Wei Sen Zhang, *PhD* ^1^, Ya Li Jin, *MPhil* ^1^, Feng Zhu, *PhD* ^1^, Kar Keung Cheng, *PhD* ^4^, Tai Hing Lam, *MD* ^1,3^

1 Guangzhou No.12 Hospital, Guangzhou 510620, China

2 School of Public Health, Sun Yat-Sen University, Guangzhou, China

3 School of Public Health, the University of Hong Kong, Hong Kong

4 Institute of Applied Health Research, University of Birmingham, Birmingham, UK

Corresponding author: Professor Lin Xu

School of Public Health, Sun Yat-sen University

Guangzhou, Guangdong Province, China

**Tel:** (86) 20-8733-5523

**Email:** [xulin27@mail.sysu.edu.cn](mailto:xulin27@mail.sysu.edu.cn)

**Supplementary Information**

Supplementary Table 1: ICD-10 definition of analysed endpoints

| **Endpoint** | **ICD-10 Definition** | **Notes** |
| --- | --- | --- |
| Cardiovascular disease | I00-I99 | Excluding I26-I27 |
| Stroke | I60-I69 |  |
| IHD | I20-I25 |  |
| Cancer | C00-C96 |  |
| Liver cancer | C22 |  |
| Colorectal cancer | C18-20 |  |
| Respiratory disease | J00-J99 | Excluding neoplastic disease |

IHD; ischaemic heart disease

Supplementary Table 2. Adjusted hazards ratio (AHRs) for all-cause and cause-specific mortality by body mass index (BMI) groups additionally adjusting for waist circumference, recruited during 2003-8 and followed up till December 2017, after excluding smokers and those with poor health.

|  | BMI. Kg/m^2^ | | | | | |
| --- | --- | --- | --- | --- | --- | --- |
|  | <18.5 | 18.5 to <20 | 20 to <22.5 | 22.5 to <25 | 25 to <27.5 | ≥27.5 |
| All-cause |  |  |  |  |  |  |
| AHR^a^ (95% CI) | 1.67 (1.32-2.1)*** | 1.4 (1.15-1.71)** | 1.00 | 0.95 (0.82-1.09) | 0.92 (0.77-1.09) | 0.97 (0.78-1.21) |
| All cancer |  |  |  |  |  |  |
| AHR^a^ (95% CI) | 1.5 (1.01-2.21)* | 1.24 (0.89-1.73) | 1.00 | 0.94 (0.75-1.18) | 0.8 (0.6-1.06) | 0.95 (0.67-1.36) |
| Colorectal cancer |  |  |  |  |  |  |
| AHR^a^ (95% CI) | 2.88 (1.28-6.47)* | 0.48 (0.14-1.61) | 1.00 | 0.62 (0.34-1.11) | 0.66 (0.33-1.29) | 0.74 (0.32-1.72) |
| Cardiovascular disease |  |  |  |  |  |  |
| AHR^b^ (95% CI) | 1.63 (1.09-2.43)* | 1.68 (1.22-2.31)** | 1.00 | 0.99 (0.78-1.27) | 1.03 (0.77-1.38) | 1.08 (0.74-1.56) |

CI: confidence interval;

a: Adjusted for age, sex, occupation, personal income, physical activity, alcohol use, smoking, self-rated health and waist circumference

*: P<0.05; **: P<0.01; ***: P<0.001

Supplementary Table 3. Adjusted hazards ratio (AHRs) for all-cause and cause-specific mortality by body mass index (BMI) groups in those aged <85 years, recruited during 2003-8 and followed up till December 2017, after excluding smokers and those with poor health.

|  | BMI. Kg/m^2^ | | | | | |
| --- | --- | --- | --- | --- | --- | --- |
|  | <18.5 | 18.5 to <20 | 20 to <22.5 | 22.5 to <25 | 25 to <27.5 | ≥27.5 |
| All-cause |  |  |  |  |  |  |
| AHR^a^ (95% CI) | 1.59 (1.26-2.01)*** | 1.37 (1.12-1.66)** | 1.00 | 0.95 (0.82-1.09) | 0.92 (0.77-1.09) | 0.98 (0.79-1.22) |
| All cancer |  |  |  |  |  |  |
| AHR^a^ (95% CI) | 1.47 (0.99-2.18) | 1.23 (0.88-1.71) | 1.00 | 0.94 (0.75-1.18) | 0.8 (0.6-1.06) | 0.96 (0.67-1.36) |
| Colorectal cancer |  |  |  |  |  |  |
| AHR^a^ (95% CI) | 2.85 (1.27-6.42)* | 0.48 (0.14-1.6) | 1.00 | 0.62 (0.34-1.11) | 0.65 (0.33-1.29) | 0.74 (0.32-1.72) |
| Cardiovascular disease |  |  |  |  |  |  |
| AHR^b^ (95% CI) | 1.51 (1-2.26)* | 1.6 (1.16-2.2)** | 1.00 | 0.98 (0.77-1.26) | 1.03 (0.77-1.37) | 1.09 (0.75-1.57) |

CI: confidence interval;

a: Adjusted for age, sex, occupation, personal income, physical activity, alcohol use, smoking, self-rated health and waist circumference

*: P<0.05; **: P<0.01; ***: P<0.001

Supplementary Table 4. Adjusted hazards ratio (AHRs) for all-cause and cause-specific mortality by adiposity using conventional Chinese-specific cut-offs for general obesity status, with and without exclusion for ever smokers and those with poor health status.

|  |  |  | General adiposity |  | Central adiposity | |
| --- | --- | --- | --- | --- | --- | --- |
|  | Underweight  (BMI <18.5kg/m^2^) | Normal^c^  (18.5≤BMI<25 kg/m^2^) | Overweight  (25≤BMI<27.5 kg/m^2^) | Obese  (BMI≥27.5 kg/m^2^) | Normal | Obese  (WC≥90cm for Men;  ≥80cm for women) |
| Person years | 14838 | 212227 | 73110 | 43986 | 224488 | 119672 |
| All-cause |  |  |  |  |  |  |
| No. of deaths | 261 | 2382 | 796 | 530 | 2498 | 1471 |
| AHR^a^ (95% CI) | 1.25 (1.1-1.43)** | 1.00 | 1.03 (0.95-1.11) | 1.17 (1.07-1.29)** | 1.00 | 1.13 (1.06-1.21)*** |
| AHR^b^ (95% CI) | 1.28 (1.05-1.56)* | 1.00 | 1.07 (0.95-1.21) | 1.30 (1.13-1.5)*** | 1.00 | 1.18 (1.07-1.3)** |
| All cancer |  |  |  |  |  |  |
| No. of deaths | 89 | 914 | 281 | 202 | 971 | 515 |
| AHR^a^ (95% CI) | 1.19 (0.96-1.49) | 1.00 | 0.95 (0.83-1.09) | 1.19 (1.02-1.39)* | 1.00 | 1.09 (0.98-1.23) |
| AHR^b^ (95% CI) | 1.16 (0.82-1.63) | 1.00 | 0.96 (0.78-1.18) | 1.32 (1.06-1.65)* | 1.00 | 1.12 (0.95-1.32) |
| Breast cancer |  |  |  |  |  |  |
| No. of deaths | 2 | 37 | 6 | 11 | 30 | 26 |
| AHR^a^ (95% CI) | 0.76 (0.18-3.15) | 1.00 | 0.5 (0.21-1.18) | 1.43 (0.73-2.8) | 1.00 | 1.32 (0.77-2.25) |
| AHR^b^ (95% CI) | 0.96 (0.23-4.08) | 1.00 | 0.25 (0.06-1.03) | 1.22 (0.5-2.96) | 1.00 | 0.92 (0.46-1.83) |
| Liver cancer |  |  |  |  |  |  |
| No. of deaths | 11 | 120 | 28 | 22 | 122 | 59 |
| AHR^a^ (95% CI) | 0.96 (0.5-1.84) | 1.00 | 0.7 (0.46-1.07) | 1.04 (0.66-1.64) | 1.00 | 1.11 (0.8-1.54) |
| AHR^b^ (95% CI) | 0.87 (0.31-2.43) | 1.00 | 0.91 (0.51-1.63) | 1.19 (0.62-2.29) | 1.00 | 1.11 (0.7-1.77) |
| Colorectal cancer |  |  |  |  |  |  |
| No. of deaths | 12 | 114 | 42 | 32 | 126 | 74 |
| AHR^a^ (95% CI) | 1.37 (0.75-2.5) | 1.00 | 1.16 (0.81-1.66) | 1.52 (1.02-2.26)* | 1.00 | 1.11 (0.82-1.51) |
| AHR^b^ (95% CI) | 2.33 (1.18-4.61)* | 1.00 | 1.24 (0.75-2.07) | 1.86 (1.09-3.18)* | 1.00 | 1.04 (0.69-1.59) |
| Cardiovascular disease |  |  |  |  |  |  |
| No. of deaths | 74 | 825 | 305 | 206 | 835 | 575 |
| AHR^a^ (95% CI) | 0.98 (0.77-1.25) | 1.00 | 1.13 (0.99-1.30) | 1.28 (1.1-1.5)** | 1.00 | 1.25 (1.11-1.4)*** |
| AHR^b^ (95% CI) | 1.21 (0.86-1.71) | 1.00 | 1.14 (0.93-1.39) | 1.35 (1.07-1.7)* | 1.00 | 1.19 (1.01-1.4)* |
| IHD |  |  |  |  |  |  |
| No. of deaths | 42 | 355 | 141 | 92 | 366 | 264 |
| AHR^a^ (95% CI) | 1.29 (0.93-1.8) | 1.00 | 1.22 (1.00-1.49)* | 1.35 (1.07-1.71)* | 1.00 | 1.33 (1.12-1.58)** |
| AHR^b^ (95% CI) | 1.57 (0.96-2.57) | 1.00 | 1.21 (0.87-1.67) | 1.39 (0.96-2.02) | 1.00 | 1.2 (0.93-1.57) |
| Stroke |  |  |  |  |  |  |
| No. of deaths | 25 | 332 | 122 | 77 | 337 | 219 |
| AHR^a^ (95% CI) | 0.84 (0.56-1.28) | 1.00 | 1.13 (0.91-1.39) | 1.2 (0.93-1.54) | 1.00 | 1.17 (0.98-1.41) |
| AHR^b^ (95% CI) | 1.13 (0.66-1.92) | 1.00 | 1.00 (0.73-1.37) | 1.3 (0.91-1.85) | 1.00 | 1.11 (0.86-1.43) |
| Respiratory disease |  |  |  |  |  |  |
| No. of deaths | 53 | 266 | 86 | 55 | 305 | 155 |
| AHR^a^ (95% CI) | 2.19 (1.62-2.97)*** | 1.00 | 0.98 (0.76-1.26) | 1.15 (0.85-1.53) | 1.00 | 1.04 (0.85-1.28) |
| AHR^b^ (95% CI) | 1.76 (1.04-2.99)* | 1.00 | 1.01 (0.68-1.49) | 1.44 (0.94-2.21) | 1.00 | 1.26 (0.93-1.72) |
| Diabetes |  |  |  |  |  |  |
| No. of deaths | 3 | 49 | 14 | 12 | 42 | 36 |
| AHR^a^ (95% CI) | 0.79 (0.24-2.54) | 1.00 | 0.85 (0.47-1.55) | 1.22 (0.64-2.3) | 1.00 | 1.27 (0.79-2.05) |
| AHR^b^ (95% CI) | 1.48 (0.18-11.87) | 1.00 | 2.89 (1.08-7.73)* | 4.6 (1.66-12.77)** | 1.00 | 2.83 (1.17-6.83)* |

CI: confidence interval; IHD: ischemic heart disease

a: Adjusted for age, sex, occupation, personal income, physical activity, alcohol use, smoking and self-rated health

b: Based on adjusted model a, after excluding ever smokers and those with poor health status.

C: Reference group: normal BMI

*: P<0.05; **: P<0.01; ***: P<0.001

Supplementary Table 5 Baseline characteristic of 29,981 participants aged 50+ in the Guangzhou Biobank Cohort Study first examined in 2003 to 2008 and followed up until January 2016, with no exclusion

|  | BMI, kg/m^2^ | | | | | | |  |
| --- | --- | --- | --- | --- | --- | --- | --- | --- |
|  | <18.5 | 18.5 to <20 | 20 to <22.5 | 22.5 to <25 | 25 to <27.5 | 27.5 to <30 | 30+ | P value |
| Number of participants (row %) | 1326 (4.4) | 2172 (7.2) | 7099 (23.7) | 9205 (30.7) | 6341 (21.1) | 2694 (9) | 1151 (3.8) |  |
| Age, years, mean (SD) | 63.5 (7.5) | 62.5 (7.4) | 61.7 (7.3) | 61.9 (7) | 62 (6.9) | 62 (7) | 61.8 (6.9) | <0.001 |
| Sex, % men | 33.0 | 30.8 | 27.4 | 28.7 | 27.5 | 23.8 | 18.1 | <0.001 |
| Education, % Primary or below | 44.1 | 40.3 | 39.7 | 41.3 | 45.1 | 48.7 | 53.3 | <0.001 |
| Occupation, % Manual | 63.0 | 60.4 | 59.8 | 60.2 | 62.1 | 63.1 | 65.4 | <0.001 |
| Family income (CNY/year), % <30,000 | 38.9 | 39.8 | 38.5 | 36.6 | 37.9 | 39.4 | 36.9 | <0.001 |
| Physical activity, % Active | 47.7 | 51.9 | 51.8 | 51.4 | 51.0 | 49.0 | 47.7 | 0.003 |
| Alcohol use, % Current | 20.2 | 24.6 | 23.7 | 25.0 | 24.9 | 24.3 | 22.2 | 0.003 |
| Smoking, % Current | 18.2 | 15.3 | 11.0 | 9.6 | 8.1 | 7.2 | 5.2 | <0.001 |
| Self-rated health, % good | 72.1 | 79.2 | 82.5 | 83.7 | 83.4 | 81.8 | 81.4 | <0.001 |
| Health status, % good | 86.9 | 85.4 | 83.2 | 79.7 | 78.1 | 74.7 | 71.2 | <0.001 |
|  | Waist circumference, cm | | | | | | |  |
|  | <70 for M  <65 for F | 70 to <75 for M  65 to <70 for F | 75 to <80 for M  70 to <75 for F | 80 to <85 for M  75 to <80 for F | 85 to <90 for M  80 to <85 for F | 90 to <95 for M  85 to <90 for F | 95+ for M  90+ for F | P value |
| Number of participants (row %) | 2062 (6.9) | 3485 (11.6) | 5825 (19.4) | 6735 (22.5) | 5804 (19.4) | 3459 (11.5) | 2618 (8.7) | - |
| Age, years, mean (SD) | 61.5 (7.4) | 60.6 (7.2) | 61.1 (7.1) | 61.7 (7) | 62.5 (6.9) | 63.3 (6.8) | 64 (7) | <0.001 |
| Sex, % men | 37.6 | 28.9 | 25.8 | 27.9 | 26.5 | 27.7 | 23.9 | <0.001 |
| Education, % Primary or below | 35.4 | 34.9 | 37.4 | 40.3 | 47.3 | 51.4 | 57.4 | <0.001 |
| Occupation, % Manual | 58.1 | 57.4 | 58.9 | 60.4 | 62.6 | 64.8 | 66.7 | <0.001 |
| Family income (CNY/year), % <30,000 | 37.9 | 38.1 | 36.8 | 36.6 | 37.4 | 40.3 | 41.6 | <0.001 |
| Physical activity, % Active | 48.7 | 50.7 | 51.6 | 51.3 | 50.6 | 50.4 | 52.2 | <0.001 |
| Alcohol use, % Current | 25.5 | 25.7 | 25.4 | 24.3 | 23.9 | 22.6 | 21.8 | 0.004 |
| Smoking, % Current | 18.1 | 12.3 | 9.8 | 9.4 | 8.9 | 8.6 | 7.5 | <0.001 |
| Self-rated health, % poor | 76.9 | 82.5 | 82.9 | 83.6 | 82.8 | 81.9 | 80.4 | <0.001 |
| Health status, % good | 88.1 | 86.1 | 83.3 | 80.0 | 77.5 | 75.1 | 71.9 | <0.001 |

:

Supplementary Table 6. Adjusted hazards ratio (AHRs) for all-cause and cause-specific mortality by body mass index (BMI) in 29,981 participants recruited during 2003-8 and followed up till December 2017, with no exclusion.

|  | BMI. Kg/m^2^ | | | | | | BMI ≥22.5 kg/m^2^ |
| --- | --- | --- | --- | --- | --- | --- | --- |
|  | <18.5 | 18.5 to <20 | 20 to <22.5^b^ | 22.5 to <25 | 25 to <27.5 | ≥27.5 | Per 5 kg/m2 |
| Person years | 14838 | 24463 | 81435 | 106329 | 73110 | 43986 | 344161 |
| All-cause |  |  |  |  |  |  |  |
| No. of deaths | 261 | 357 | 894 | 1131 | 796 | 530 | 3969 |
| AHR^a^ (95% CI) | 1.28 (1.11-1.47)** | 1.18 (1.04-1.34)** | 1.00 | 0.98 (0.9-1.08) | 1.04 (0.95-1.15) | 1.19 (1.07-1.33)** | 1.20 (1.11-1.3)*** |
| All cancer |  |  |  |  |  |  |  |
| No. of deaths | 89 | 131 | 346 | 437 | 281 | 202 | 1,486 |
| AHR^a^ (95% CI) | 1.21 (0.96-1.54) | 1.16 (0.95-1.43) | 1.00 | 0.99 (0.86-1.15) | 0.96 (0.82-1.13) | 1.21 (1.02-1.45)* | 1.24 (1.09-1.4)** |
| Liver cancer |  |  |  |  |  |  |  |
| No. of deaths | 11 | 13 | 44 | 63 | 28 | 22 | 181 |
| AHR^a^ (95% CI) | 1.03 (0.51-2.06) | 0.95 (0.51-1.78) | 1.00 | 1.16 (0.78-1.73) | 0.75 (0.46-1.22) | 1.11 (0.66-1.87) | 0.87 (0.57-1.31) |
| Colorectal cancer |  |  |  |  |  |  |  |
| No. of deaths | 12 | 13 | 43 | 58 | 42 | 32 | 200 |
| AHR^a^ (95% CI) | 1.38 (0.72-2.63) | 0.97 (0.52-1.82) | 1.00 | 1.01 (0.67-1.52) | 1.17 (0.76-1.8) | 1.53 (0.96-2.43) | 1.56 (1.17-2.09)** |
| Cardiovascular disease |  |  |  |  |  |  |  |
| No. of deaths | 74 | 122 | 304 | 399 | 305 | 206 | 1,410 |
| AHR^a^ (95% CI) | 1.02 (0.79-1.33) | 1.2 (0.97-1.48) | 1.00 | 1.04 (0.89-1.21) | 1.19 (1.01-1.39)* | 1.34 (1.12-1.6)** | 1.24 (1.09-1.4)** |
| IHD |  |  |  |  |  |  |  |
| No. of deaths | 42 | 56 | 124 | 175 | 141 | 92 | 630 |
| AHR^a^ (95% CI) | 1.44 (1-2.06)* | 1.35 (0.99-1.86) | 1.00 | 1.13 (0.9-1.43) | 1.36 (1.06-1.73)* | 1.50 (1.14-1.97)** | 1.29 (1.08-1.55)** |
| Stroke |  |  |  |  |  |  |  |
| No. of deaths | 25 | 51 | 125 | 156 | 122 | 77 | 556 |
| AHR^a^ (95% CI) | 0.86 (0.56-1.34) | 1.22 (0.88-1.7) | 1.00 | 0.98 (0.78-1.25) | 1.15 (0.89-1.48) | 1.22 (0.91-1.63) | 1.20 (0.97-1.47) |
| Respiratory disease |  |  |  |  |  |  |  |
| No. of deaths | 53 | 43 | 97 | 126 | 86 | 55 | 460 |
| AHR^a^ (95% CI) | 2.25 (1.6-3.16)*** | 1.14 (0.79-1.66) | 1.00 | 1.01 (0.77-1.32) | 1 (0.75-1.35) | 1.17 (0.84-1.63) | 1.14 (0.9-1.44) |
| Diabetes |  |  |  |  |  |  |  |
| No. of deaths | 3 | 9 | 20 | 20 | 14 | 12 | 78 |
| AHR^a^ (95% CI) | 0.7 (0.21-2.36) | 1.44 (0.66-3.17) | 1.00 | 0.66 (0.35-1.26) | 0.75 (0.38-1.49) | 1.07 (0.52-2.2) | 1.47 (0.86-2.53) |

CI: confidence interval; IHD: ischemic heart disease

a: Adjusted for age, sex, occupation, personal income, physical activity, alcohol use, smoking and self-rated health

b: Reference group: BMI of 20 to <22.5

*: P<0.05; **: P<0.01; ***: P<0.001

Supplementary Table 7. Adjusted hazards ratio (HRs) for all-cause and cause-specific mortality by waist circumference, recruited during 2003-8 and followed up till December 2017, with no exclusion.

|  | Waist circumference, cm | | | | | | | 78+ for M and  72+ for M;  Per 10 cm |
| --- | --- | --- | --- | --- | --- | --- | --- | --- |
|  | <70 for M  <65 for F | 70 to <75 for M  65 to <70 for F | 75 to <80 for M  70 to <75 for F | 80 to <85 for M  75 to <80 for F | 85 to <90 for M  80 to <85 for F | 90 to <95 for M  85 to <90 for F | 95+ for M  90+ for F |  |
| Person years | 23093 | 39489 | 66803 | 77930 | 67039 | 39870 | 29936 | 344161 |
| All-cause |  |  |  |  |  |  |  |  |
| No. of deaths | 304 | 410 | 664 | 770 | 794 | 547 | 480 | 3969 |
| AHR^a^ (95% CI) | 1.11 (0.96-1.27) | 1.05 (0.92-1.19) | Ref. | 0.94 (0.85-1.05) | 1.08 (0.97-1.2) | 1.14 (1.02-1.28)* | 1.27 (1.13-1.44)*** | 1.14 (1.08-1.19)*** |
| All cancer |  |  |  |  |  |  |  |  |
| No. of deaths | 115 | 170 | 258 | 295 | 284 | 181 | 183 | 1,486 |
| AHR^a^ (95% CI) | 1.12 (0.89-1.41) | 1.13 (0.92-1.38) | Ref. | 0.97 (0.81-1.15) | 1.07 (0.9-1.27) | 1.07 (0.88-1.3) | 1.41 (1.16-1.71)** | 1.14 (1.05-1.23)** |
| Liver cancer |  |  |  |  |  |  |  |  |
| No. of deaths | 13 | 17 | 28 | 44 | 40 | 18 | 21 | 181 |
| AHR^a^ (95% CI) | 1.01 (0.51-2) | 0.93 (0.49-1.75) | Ref. | 1.32 (0.82-2.14) | 1.41 (0.86-2.3) | 0.98 (0.54-1.79) | 1.5 (0.84-2.66) | 1.03 (0.82-1.28) |
| Colorectal cancer |  |  |  |  |  |  |  |  |
| No. of deaths | 9 | 20 | 40 | 37 | 38 | 25 | 31 | 200 |
| AHR^a^ (95% CI) | 0.62 (0.3-1.29) | 0.85 (0.49-1.48) | Ref. | 0.84 (0.54-1.31) | 0.9 (0.57-1.42) | 0.88 (0.52-1.48) | 1.55 (0.96-2.5) | 1.19 (0.97-1.46) |
| Cardiovascular disease |  |  |  |  |  |  |  |  |
| No. of deaths | 88 | 121 | 231 | 269 | 304 | 216 | 181 | 1,410 |
| AHR^a^ (95% CI) | 0.89 (0.69-1.15) | 0.89 (0.72-1.12) | Ref. | 0.93 (0.78-1.12) | 1.12 (0.94-1.33) | 1.24 (1.03-1.49)* | 1.28 (1.05-1.56)* | 1.16 (1.08-1.26)*** |
| IHD |  |  |  |  |  |  |  |  |
| No. of deaths | 38 | 53 | 108 | 117 | 125 | 100 | 89 | 630 |
| AHR^a^ (95% CI) | 0.81 (0.55-1.19) | 0.84 (0.6-1.16) | Ref. | 0.86 (0.66-1.12) | 0.99 (0.76-1.28) | 1.22 (0.92-1.6) | 1.4 (1.05-1.85)* | 1.21 (1.08-1.36)** |
| Stroke |  |  |  |  |  |  |  |  |
| No. of deaths | 41 | 48 | 90 | 107 | 119 | 84 | 67 | 556 |
| AHR^a^ (95% CI) | 1.08 (0.74-1.58) | 0.93 (0.65-1.31) | Ref. | 0.95 (0.71-1.25) | 1.11 (0.84-1.47) | 1.24 (0.92-1.67) | 1.17 (0.85-1.61) | 1.13 (1.00-1.28) |
| Respiratory disease |  |  |  |  |  |  |  |  |
| No. of deaths | 51 | 42 | 76 | 91 | 83 | 59 | 58 | 460 |
| AHR^a^ (95% CI) | 1.54 (1.07-2.2)* | 0.87 (0.59-1.28) | Ref. | 0.93 (0.68-1.26) | 0.93 (0.68-1.28) | 1.02 (0.72-1.43) | 1.24 (0.89-1.75) | 1.12 (0.97-1.29) |
| Diabetes |  |  |  |  |  |  |  |  |
| No. of deaths | 6 | 7 | 16 | 10 | 14 | 14 | 11 | 78 |
| AHR^a^ (95% CI) | 1.05 (0.41-2.7) | 0.79 (0.33-1.93) | Ref. | 0.46 (0.2-1.04) | 0.73 (0.36-1.51) | 0.94 (0.44-1.99) | 1.04 (0.48-2.25) | 1.13 (0.81-1.58) |

CI: confidence interval; IHD: ischemic heart disease

a: Adjusted for age, sex, occupation, personal income, physical activity, alcohol use, smoking and self-rated health

*: P<0.05; **: P<0.01; ***: P<0.001

Supplementary Table 8. Adjusted hazards ratio (AHRs) for all-cause and cause-specific mortality by waist circumference, recruited during 2003-8 and followed up till December 2017, with and without exclusion.

|  | Waist circumference, cm | | | | | | | Per 10 cm |
| --- | --- | --- | --- | --- | --- | --- | --- | --- |
|  | <70 for M  <65 for F | 70 to <75 for M  65 to <70 for F | 75 to <80 for M  70 to <75 for F | 80 to <85 for M  75 to <80 for F | 85 to <90 for M  80 to <85 for F | 90 to <95 for M  85 to <90 for F | 95+ for M  90+ for F |  |
| Person years | 23093 | 39489 | 66803 | 77930 | 67039 | 39870 | 29936 | 344161 |
| All-cause |  |  |  |  |  |  |  |  |
| No. of deaths | 304 | 410 | 664 | 770 | 794 | 547 | 480 | 3969 |
| AHR^a^ (95% CI) | Ref. | 0.94 (0.81-1.1) | 0.9 (0.78-1.04) | 0.97 (0.85-1.12) | 0.97 (0.85-1.12) | 1.03 (0.89-1.19) | 1.15 (0.99-1.33) | 1.06 (1.02-1.1)** |
| AHR^b^ (95% CI) | Ref. | 1.01 (0.79-1.29) | 0.99 (0.79-1.24) | 1.06 (0.85-1.32) | 1.06 (0.85-1.32) | 1.15 (0.91-1.45) | 1.39 (1.1-1.76)** | 1.11 (1.05-1.17)*** |
| All cancer |  |  |  |  |  |  |  |  |
| No. of deaths | 115 | 170 | 258 | 295 | 284 | 181 | 183 | 1,486 |
| AHR^a^ (95% CI) | Ref. | 1.02 (0.8-1.3) | 0.9 (0.72-1.13) | 0.96 (0.77-1.2) | 0.96 (0.77-1.2) | 0.96 (0.75-1.22) | 1.27 (1.00-1.62)* | 1.05 (0.99-1.12) |
| AHR^b^ (95% CI) | Ref. | 1.15 (0.77-1.73) | 1.11 (0.76-1.61) | 1.08 (0.74-1.57) | 1.08 (0.74-1.57) | 1.21 (0.82-1.8) | 1.78 (1.2-2.63)** | 1.13 (1.03-1.23)** |
| Liver cancer |  |  |  |  |  |  |  |  |
| No. of deaths | 13 | 17 | 28 | 44 | 40 | 18 | 21 | 181 |
| AHR^a^ (95% CI) | Ref. | 0.93 (0.43-1.98) | 1 (0.5-1.97) | 1.4 (0.73-2.69) | 1.4 (0.73-2.69) | 0.93 (0.44-1.95) | 1.5 (0.73-3.08) | 1.1 (0.93-1.3) |
| AHR^b^ (95% CI) | Ref. | 0.8 (0.22-3.01) | 1.05 (0.33-3.31) | 1.76 (0.6-5.19) | 1.76 (0.6-5.19) | 1.21 (0.37-3.97) | 1.85 (0.57-5.97) | 1.17 (0.91-1.49) |
| Colorectal cancer |  |  |  |  |  |  |  |  |
| No. of deaths | 9 | 20 | 40 | 37 | 38 | 25 | 31 | 200 |
| AHR^a^ (95% CI) | Ref. | 1.38 (0.62-3.05) | 1.61 (0.78-3.34) | 1.29 (0.62-2.67) | 1.46 (0.7-3.03) | 1.43 (0.66-3.1) | 2.51 (1.19-5.3)* | 1.2 (1.02-1.4)* |
| AHR^b^ (95% CI) | Ref. | 1.4 (0.43-4.55) | 1.84 (0.63-5.36) | 1.67 (0.57-4.86) | 1.5 (0.5-4.47) | 1.76 (0.57-5.49) | 3.36 (1.11-10.1)* | 1.26 (1.01-1.58)* |
| Cardiovascular disease |  |  |  |  |  |  |  |  |
| No. of deaths | 88 | 121 | 231 | 269 | 304 | 216 | 181 | 1,410 |
| AHR^a^ (95% CI) | Ref. | 0.99 (0.75-1.32) | 1.12 (0.87-1.44) | 1.05 (0.82-1.34) | 1.28 (1-1.63) | 1.39 (1.07-1.8)* | 1.44 (1.1-1.87)** | 1.14 (1.08-1.21)*** |
| AHR^b^ (95% CI) | Ref. | 1.04 (0.68-1.59) | 1.13 (0.76-1.67) | 0.85 (0.57-1.26) | 1.25 (0.85-1.82) | 1.23 (0.82-1.83) | 1.39 (0.92-2.09) | 1.12 (1.02-1.22)* |
| IHD |  |  |  |  |  |  |  |  |
| No. of deaths | 38 | 53 | 108 | 117 | 125 | 100 | 89 | 630 |
| AHR^a^ (95% CI) | Ref. | 1.02 (0.66-1.56) | 1.24 (0.84-1.81) | 1.06 (0.73-1.56) | 1.24 (0.85-1.81) | 1.5 (1.02-2.22)* | 1.72 (1.16-2.56)** | 1.18 (1.08-1.29)*** |
| AHR^b^ (95% CI) | Ref. | 0.92 (0.48-1.75) | 0.99 (0.55-1.78) | 0.71 (0.39-1.28) | 0.91 (0.5-1.62) | 1.08 (0.59-1.97) | 1.36 (0.74-2.5) | 1.1 (0.96-1.27) |
| Stroke |  |  |  |  |  |  |  |  |
| No. of deaths | 41 | 48 | 90 | 107 | 119 | 84 | 67 | 556 |
| AHR^a^ (95% CI) | Ref. | 0.84 (0.55-1.28) | 0.92 (0.63-1.35) | 0.87 (0.6-1.26) | 1.04 (0.72-1.5) | 1.14 (0.78-1.68) | 1.08 (0.72-1.61) | 1.09 (0.99-1.2) |
| AHR^b^ (95% CI) | Ref. | 0.84 (0.46-1.55) | 0.97 (0.56-1.67) | 0.65 (0.37-1.13) | 1.15 (0.68-1.93) | 0.82 (0.46-1.46) | 1.04 (0.58-1.88) | 1.05 (0.92-1.2) |
| Respiratory disease |  |  |  |  |  |  |  |  |
| No. of deaths | 51 | 42 | 76 | 91 | 83 | 59 | 58 | 460 |
| AHR^a^ (95% CI) | Ref. | 0.55 (0.36-0.84)** | 0.64 (0.44-0.91)* | 0.61 (0.43-0.86)** | 0.6 (0.42-0.86)** | 0.67 (0.45-0.98)* | 0.8 (0.55-1.18) | 0.98 (0.89-1.09) |
| AHR^b^ (95% CI) | Ref. | 0.59 (0.28-1.24) | 0.8 (0.43-1.51) | 0.67 (0.36-1.25) | 0.77 (0.41-1.44) | 0.98 (0.51-1.87) | 1.23 (0.64-2.36) | 1.12 (0.95-1.33) |
| Diabetes |  |  |  |  |  |  |  |  |
| No. of deaths | 6 | 7 | 16 | 10 | 14 | 14 | 11 | 78 |
| AHR^a^ (95% CI) | Ref. | 0.75 (0.25-2.25) | 0.95 (0.37-2.44) | 0.44 (0.16-1.24) | 0.7 (0.27-1.83) | 0.89 (0.33-2.4) | 0.99 (0.36-2.7) | 1.03 (0.8-1.33) |
| AHR^b^ (95% CI) | Ref. | - | 0.69 (0.12-3.78) | 0.27 (0.04-1.95) | 0.82 (0.16-4.15) | 0.85 (0.15-4.71) | 1.63 (0.32-8.33) | 1.72 (1.11-2.66)* |

CI: confidence interval; IHD: ischemic heart disease

a: Adjusted for age, sex (except for breast cancer mortality), occupation, personal income, physical activity, alcohol use, smoking and self-rated health

b: Based on adjusted model a, after excluding smokers and those with poor health status.

*: P<0.05; **: P<0.01; ***: P<0.001

Supplementary Table 9. Adjusted hazards ratio (HRs) for all-cause and cardiovascular disease mortality by waist circumference groups, after excluding smokers and those with poor health status

|  | All-cause mortality | | Cardiovascular disease mortality | |
| --- | --- | --- | --- | --- |
| Waist circumference, cm | AHR^a^ (95% CI) | AHR^b^ (95% CI) | AHR^a^ (95% CI) | AHR^b^ (95% CI) |
| 60 to <70 for M; 60 to <65 for F | 1.00 | 1.00 | 1.00 | 1.00 |
| 70 to <75 for M; 65 to <70 for F | 1.03 (0.79-1.34) | 1.11 (0.78-1.57) | 1.02 (0.65-1.60) | 1.15 (0.61-2.16) |
| 75 to <80 for M; 70 to <75 for F | 1.01 (0.79-1.29) | 1.08 (0.77-1.51) | 1.10 (0.73-1.68) | 1.29 (0.71-2.34) |
| 80 to <85 for M; 75 to <80 for F | 0.90 (0.71-1.15) | 0.99 (0.72-1.38) | 0.83 (0.55-1.27) | 0.99 (0.54-1.8) |
| 85 to <90 for M; 80 to <85 for F | 1.09 (0.86-1.38) | 1.20 (0.86-1.66) | 1.22 (0.81-1.84) | 1.44 (0.79-2.6) |
| 90 to <95 for M; 85 to <90 for F | 1.17 (0.91-1.50) | 1.30 (0.93-1.82) | 1.20 (0.79-1.84) | 1.42 (0.77-2.6) |
| 95+ for M; 90+ for F | 1.42 (1.10-1.83)** | 1.57 (1.12-2.21)** | 1.36 (0.88-2.11) | 1.6 (0.87-2.96) |

CI: confidence interval

a: Adjusted for age, sex, occupation, personal income, physical activity, alcohol use, smoking and self-rated health.

b: Adjusted for age, sex, occupation, personal income, physical activity, alcohol use, smoking and self-rated health, after excluding BMI<18.5 kg/m^2^.

*: P<0.05; **: P<0.01; ***: P<0.001

Supplementary Table 10. Adjusted hazards ratio (AHRs) for all-cause and cause-specific mortality by waist circumference groups, after excluding smokers and those with poor health status and additionally adjusting for BMI

|  | Waist circumference, cm | | | | | | |
| --- | --- | --- | --- | --- | --- | --- | --- |
|  | <70 for M  <65 for F | 70 to <75 for M  65 to <70 for F | 75 to <80 for M  70 to <75 for F | 80 to <85 for M  75 to <80 for F | 85 to <90 for M  80 to <85 for F | 90 to <95 for M  85 to <90 for F | 95+ for M  90+ for F |
| All-cause |  |  |  |  |  |  |  |
| AHR^a^ (95% CI) | Ref. (1.0) | 1.05 (0.82-1.35) | 1.06 (0.84-1.35) | 0.98 (0.76-1.26) | 1.21 (0.93-1.59) | 1.35 (1-1.82) | 1.71 (1.22-2.39)** |
| All cancer |  |  |  |  |  |  |  |
| AHR^a^ (95% CI) | Ref. (1.0) | 1.21 (0.8-1.82) | 1.19 (0.8-1.78) | 1.21 (0.8-1.85) | 1.24 (0.79-1.95) | 1.44 (0.87-2.38) | 2.21 (1.27-3.86)** |
| Colorectal cancer |  |  |  |  |  |  |  |
| AHR^a^ (95% CI) | Ref. (1.0) | 1.43 (0.43-4.73) | 1.9 (0.62-5.86) | 1.76 (0.54-5.73) | 1.6 (0.45-5.69) | 1.91 (0.48-7.6) | 3.73 (0.84-16.48) |
| Cardiovascular disease |  |  |  |  |  |  |  |
| AHR^a^ (95% CI) | Ref. (1.0) | 1.04 (0.67-1.61) | 1.12 (0.74-1.7) | 0.85 (0.55-1.32) | 1.24 (0.78-1.97) | 1.22 (0.74-2.04) | 1.38 (0.78-2.46) |

CI: confidence interval;

a: Adjusted for age, sex, occupation, personal income, physical activity, alcohol use, smoking, self-rated health and BMI

*: P<0.05; **: P<0.01; ***: P<0.001
